# Tunneling Atomic Force Microscopy Analysis of Supramolecular Self-Responsive Nanocomposites

**DOI:** 10.3390/polym13091401

**Published:** 2021-04-26

**Authors:** Marialuigia Raimondo, Elisa Calabrese, Wolfgang H. Binder, Philipp Michael, Sravendra Rana, Liberata Guadagno

**Affiliations:** 1 Department of Industrial Engineering, University of Salerno, Via Giovanni Paolo II, 132, 84084 Fisciano, SA, Italy; elicalabrese@unisa.it (E.C.); lguadagno@unisa.it (L.G.); 2 Macromolecular Chemistry, Institute of Chemistry, Faculty of Natural Science II, Martin Luther University Halle-Wittenberg, Von-Danckelmann-Platz 4, 06120 Halle, Germany; wolfgang.binder@chemie.uni-halle.de (W.H.B.); philipp.michael@chemie.uni-halle.de (P.M.); 3 Department of Chemistry, University of Petroleum and Energy Studies (UPES), Dehradun 248007, India; srana@ddn.upes.ac.in

**Keywords:** tunneling atomic force microscopy (TUNA), morphological analysis, self-responsive materials, self-healing nanocomposites, supramolecular interactions, multi-wall carbon nanotubes (MWCNTs), hydrogen bonding, functionalized nanofillers

## Abstract

A big step forward for composite application in the sector of structural materials is given by the use of Multi-Wall Carbon Nanotubes (MWCNTs) functionalized with hydrogen bonding moieties, such as barbiturate and thymine, to activate self-healing mechanisms and integrate additional functionalities. These materials with multiple healing properties at the same damaged site, imparted by hydrogen bonds, will also have the potential to improve material reliability, extend the service life, reduce replacement costs, and improve product safety. This revolutionary approach is obtained by integrating the non-covalent interactions coupled with the conventional covalent approach used to cross-link the polymer. The objective of this work is to characterize rubber-toughened supramolecular self-healing epoxy formulations based on unfunctionalized and functionalized MWCNTs using Tunneling Atomic Force Microscopy (TUNA). This advanced technique clearly shows the effect produced by the hydrogen bonding moieties acting as reversible healing elements by their simultaneous donor and acceptor character, and covalently linked to MWCNTs to originate self-healing nanocomposites. In particular, TUNA proved to be very effective for the morphology study of both the unfunctionalized and functionalized carbon nanotube-based conductive networks, thus providing useful insights aimed at understanding the influence of the intrinsic nature of the nanocharge on the final properties of the multifunctional composites.

## 1. Introduction

### 1.1. Overview of Smart and Multifunctional Materials

Safety and reliability for aerospace structures will be enhanced by developing and employing a condition based on a health management system for aerostructures, which is grounded on so-called “internal” health management (realized via smart aerospace materials, which will actively counteract the degradation of structures), and “external” health management (including systems of structural health monitoring and quick repair). Smart structural aerospace materials emulate biological systems, where small defects are self-healed (like blood clotting at small cuts), and larger “wounds” cause signals (as pain informs living creatures about local damage), leading to external, organized healing efforts. In this regard, the expected transition from a rigid, inert structure to smart self-responsive composite aerostructures represents the primary objective to guarantee long-lasting structural materials.

The need to design and realize multifunctional materials that meet the structural requirements is becoming more and more pressing.

Nowadays, the creation of multifunctional materials represents a real challenge since it is aimed at overcoming the criticalities connected with the use of composite materials in the aeronautical field [1,2,3,4,5,6], such as reduced electrical properties and poor damage resistance. In fact, the principal hazard associated with the use of composites is determined by the generation of micro-cracks inside the structure which are difficult to identify and repair and which can, consequently, cause catastrophic damage.

Providing these materials with the ability to self-repair is undoubtedly a winning strategy that leads consequently to the automatic recovery of their mechanical properties.

For more than a decade now, many studies have been concentrated by researchers around the world on this hot topic of great industrial interest.

They led to the production of composites with an inherent ability to repair themselves, such as to achieve a self-restorative capableness greater than 100% [7].

The recent finding has opened a doorway to various perspectives to tackle the study focused on the concept of multifunctionality in the context of epoxy structural materials. Due to great flexibility from chemical and processing viewpoints, the usefulness of epoxy resins is omnipresent in industries that have need of high performing materials.

Epoxy resins find widespread use in fiber-reinforced composites for the fabrication of structural parts of aircraft and turbines [8,9,10,11,12,13,14,15,16,17].

The majority of self-healing solutions rely on external stimuli such as temperature [18,19].

The common approach for autonomous repair of thermosetting systems [20,21,22,23,24,25,26] is the adding of molecules with an auto-repair function inside a breakable capsule or container into the polymeric matrix [27,28,29].

Upon cracking damage, the healing agents can be released to recuperate the defect. The development of microencapsulated systems (based on an epoxy precursor, a microencapsulated healing agent, and a catalytic chemical trigger within the epoxy matrix) [30,31] constitutes a significant break-through in self-healing chemistry.

Many constraints and challenges are intimately connected with the actual structural applications of self-healing materials. In fact, while soft materials allow the application of different self-healing scenarios, structural materials with limited movements of chains have a modest choice of effective self-healing mechanisms.

Ring-opening metathesis polymerization (ROMP) initiators significantly increase the production costs and limit the mechanical performance. The weak mechanical properties of microencapsulated systems are caused by the fact that the curing agents (e.g., diaminodiphenyl sulfone (DDS)) cannot be used in conjunction with initiators suitable for ROMP, and the impossibility to use curing cycles at high temperatures [20].

The too high production costs are due to the impossibility to use the initiator dispersed at a molecular level in very chemical reactive environments and the high cost of ruthenium-based initiators which must be embedded in the resins in high amount (~5 wt %) in the form of solid particles.

The problems of poor mechanical performance and high production costs of self-healing materials were solved by applying the new chemically stable ruthenium catalyst, allowing a strong reduction in manufacturing costs, a relevant enhancement in mechanical properties, and high efficiency in the self-healing function [20].

This can be realized without undergoing deactivation (using the usual industrial curing cycles up to 180 °C). Other important issues are related to the inclusion of self-repairing functions in composite structures and the manufacturing of microcapsules, which is expensive and for which no industrial production methods are available.

### 1.2. Supramolecular Chemistry Approach and Self-Responsive Advanced Composites

New supramolecular chemistry approaches which utilize numerous, reversible, and in specific cases, synergic intermolecular interactions can be taken advantage of to create new materials [32,33], resulting in a particularly beneficial way of eluding the critical issues encountered when the encapsulation of repairing agents must take place in microcapsules or vessels, and because of this, in facilitating the manufacturing processes. In this work, in the perspective of developing self-responsive composites which can open new intriguing scenarios in the field of advanced materials, structural nanocomposites endowed with self-repairing capability and high electrical conductivity were prepared using MWCNTs functionalized with hydrogen bonding moieties covalently attached to the nanofillers, giving thymine (MWCNT-t) and barbituric (MWCNT-b) acid-based ligands via a “click” reaction [34,35,36].

For these nanocomposites, the matrix employed is toughened with a rubber phase dispersed in the tetrafunctional resin. The performed functionalization leads to the formation of carbon nanotubes which act as a bridge between the rubber phase and the functional groups adhering to the walls of the carbon nanotubes.

In our previous papers, we have demonstrated that self-healing mechanisms are activated by hydrogen bonding interactions that are favored by the rubber phase well dispersed in the composite [34,35,37].

The nanocomposites based on modified carbon nanotubes, even if loaded with a low nanofiller amount of 0.5 wt %, showed a self-repair efficiency of over 50% which represents a very considerable result also by virtue of the excellent mechanical properties measured. Unfortunately, at the percentage of 0.5 wt %, the nanocomposites containing functionalized MWCNTs showed a very low electrical conductivity compared to analog nanocomposites containing unfunctionalized MWCNTs.

This reduced electrical conductivity is most likely attributable to the aspect ratio changing as a consequence of the performed functionalization. In order to obtain electrically conductive self-healing composites, the use of a higher percentage of functionalized MWCNTs (2 wt %) made it possible to reach electrical conductivity values similar to those recorded with unfunctionalized MWCNTs [34].

The self-healing mechanism integrated in multifunctional materials is characterized by high values in the glass transition temperature and storage modulus.

### 1.3. TUNA as Powerful Nanoscale Electrical Property Mapping Tool of Self-Responsive Materials

In this work, we perform a morphological characterization by TUNA with the aim of providing a mapping of the electrical conductivity of multifunctional nanocomposites based on both unfunctionalized and functionalized carbon nanotubes.

The TUNA technique proved to be particularly worthwhile in evaluating the influence of functionalization on the local conductive behavior of the nanocharge and how the latter influences the electrical percolation paths of the nanodomains within the composite. Through the TUNA, we demonstrate that, for all analyzed samples, the intrinsic electrical conductivity imparted by both unfunctionalized and functionalized carbon nanotubes is good. In particular, the formation of conductive networks is also evident for nanocomposites containing functionalized MWCNT-b and MWCNT-t at the load percentage of 0.5 wt %, which was found to be below the Electrical Percolation Threshold (EPT) [34,37].

This shows that all nanocomposites loaded with the same nanofiller percentage of 0.5 wt %, regardless of functionalization or not, always give rise to local conductive networks.

An increase in the weight concentration of the functionalized MWCNTs from 0.5 wt % up to 2 wt % determines a wider extension of the electrical conductive networks along the surface section of the sample.

In this regard, it is interesting to note how the increase in electrical conductivity of the samples containing the highest concentration of 2 wt % of nanofillers is highlighted by the ability of TUNA to detect very low currents also of the order of femtoampere (fA). Furthermore, the TUNA Current images, through the analysis of the frequency of the current changes due to nanofiller/matrix alternations, also allowed the demonstration of good dispersion of both unfunctionalized and functionalized MWCNTs. This aspect is very crucial in the view of formulating self-responsive nanocomposites for real industrial applications.

## 2. Materials and Methods

### 2.1. Materials

#### 2.1.1. Preparation of Functionalized Carbon Nanofillers

The covalent functionalization of Multi-Wall Carbon Nanotubes, which allowed chemical moieties to be attached directly to the MWCNT tubular structure by sharing at least one electron pair between the MWCNT and the introduced chemical moieties, was carried out following a procedure that has already been described in our previous papers [34,35].

**Figure 1:**
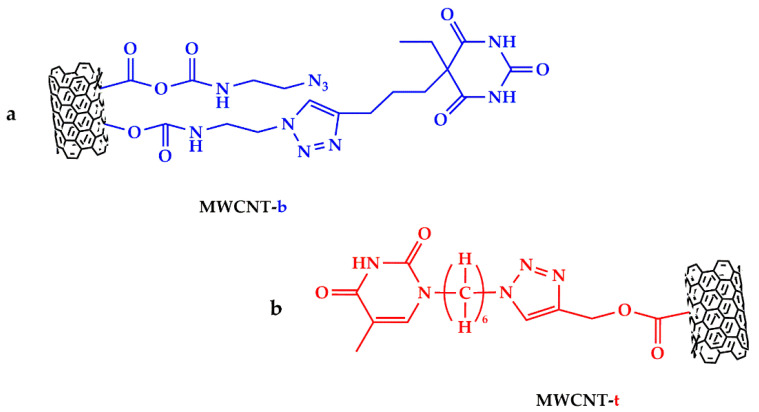
Carbon nanofillers functionalized with: (**a**) barbiturate-based ligand (MWCNT-b) and (**b**) thymine-based ligand (MWCNT-t).

In particular, in order to create strong intermolecular hydrogen bonding interactions with the functional groups of the epoxy matrix, thymine and barbiturate acid-based ligands were covalently bonded to MWCNT, giving MWCNT-t and MWCNT-b modified carbon nanofillers (Figure 1).

In fact, these ligands have N-H functional groups acting as H-bonding donor sites and C = O functional groups acting as H-bonding acceptor sites, which can interact with the C = O and O-H groups of the cured matrix, respectively, thus promoting the generation of a supramolecular network. Figure S1 (see Supplementary Electronic Materials) clearly shows the schematic representation of the supramolecular network responsible for the activation of self-healing mechanisms in the case of the sample loaded with barbiturate functionalized MWCNT-b.

From Figure S1 (see Supplementary Electronic Materials), we can see how the hydrogen bonding interactions can be activated between the functional groups of the same carbon nanofillers.

#### 2.1.2. Preparation of the Epoxy Samples

Epoxy samples loaded with both pristine and modified carbon nanotubes at different compositions have been analyzed by the TUNA technique. Their preparation procedure is described in our previous paper [34]. The formulated samples were obtained starting from a toughened epoxy matrix whose acronym is TCTBD. More specifically, TCTBD unfilled resin is composed of tetraglycidyl methylene dianiline (T) mixed with a reactive diluent, 1,4-butanedioldiglycidylether (B), in the ratio T/B of 80/20 by weight, a fluid rubber, carboxyl-terminated butadiene acrylonitrile (CT), in the percentage of 12.5 phr with respect to the epoxy precursor T, and a curing agent, 4,4′-diaminodiphenyl sulfone (D). The formation of covalent bonds due to the reaction between the COOH groups of the liquid rubber (CT) with the oxirane rings of the resin (T) was demonstrated [34].

As a result of this reaction, micrometric globular domains homogeneously dispersed in the resin were formed. In order to prepare the multifunctional nanocomposites, the starting formulation TCTBD was loaded with (a) 0.5 wt % of unfunctionalized MWCNT, (b) 0.5 wt % of the two types of functionalized MWCNT, and (c) 2 wt % of two types of functionalized MWCNT.

All the nanocomposites were solidified following a heat treatment in an oven at 125 °C for 1 h and then at 200 °C for 3 h.

### 2.2. Methods

#### 2.2.1. Fourier Transform Infrared Spectroscopy (FTIR)

A Bruker Vertex 70 FTIR-spectrophotometer (Bruker Optics Inc., Billerica, MA, USA) allowed us to carry out Fourier-transform infrared spectroscopy (FTIR) analysis in the range 4000–400 cm^−1^, with a resolution of 2 cm^−1^ (32 scans collected). The infrared spectra were acquired in absorbance.

#### 2.2.2. High-Resolution Transmission Electron Microscopy (HRTEM)

To conduct HRTEM morphological analysis, a Jeol 2010 LaBa6 microscope operating at an acceleration voltage of 200 kV was employed.

The samples were prepared by placing a droplet of ~0.1 mg mL^−1^ material solution on a copper coated carbon grid followed by air-drying.

The solution was obtained by dispersing the carbon nanofillers in ethanol by ultrasonication for about half an hour.

The fracture surface of the samples filled with 0.5 wt % MWCNT and MWCNT-b was also analyzed.

The nanocomposites underwent etching treatment [35] before being investigated by HRTEM.

#### 2.2.3. Tunneling Atomic Force Microscopy (TUNA)

Information on topography and local nanoelectrical current of the multifunctional nanocomposites was obtained by the TUNA technique operating in contact mode and using platinum-coated probes with nominal spring constants of 35 Nm^−1^ and an electrically conductive tip of 20 nm.

The TUNA module measures ultra-low currents (<1 pA) ranging from 80 fA to 120 pA circulating through the conductive tip to the investigated samples kept at a fixed DC bias. In this work, we used a DC sample bias from 1 to 2 V.

A linear current amplifier with a range of 60–120 fA detects the resulting current passing through the samples. In this way, the sample’s topography and current are measured at the same time, activating direct correlation of a sample location with its electrical properties.

It is worth noting that notably sensitive current measurements were allowed due to the noise level of the TUNA module commonly of 50 fA.

Highest resolution current mapping of the nanocomposites was obtained with the current sensitivity of the TUNA module which selects the gain referring to the output voltage of the TUNA sensor set to 1 pA/V, corresponding to the gain of 10^12^, scan rate of 0.500 Hz s^−1^, and number of pixels in X and Y (samples/line) set to 512.

The detected areas of the analyzed samples are representative of the entire supramolecular self-responsive nanocomposites because, in order to obtain electrical measurements at a nanoscale level with indisputable repeatability and reproducibility, a cantilever with a sharp tip was scanned on different areas over a sample surface so that each TUNA image reported in the manuscript was captured after verifying that the electrical response was the same at least five various scanned points.

It is worth noting that, generally, it is not enough that the tip is in contact with a conductive material but electrical contacts to the ground ensured by silver paste are also essential for the current to flow.

Thus, a current signal is obtained only if the tip during the sample contact constitutes a part of a closed electrical circuit. In this work, the nanoelectrical characterization was carried out without grounding the samples.

The TUNA results show that, even if the analyzed samples were not grounded, it is possible to detect electric current values that irrefutably prove the intrinsic electrical conductivity of the formulated nanocomposites.

**Figure 2:**
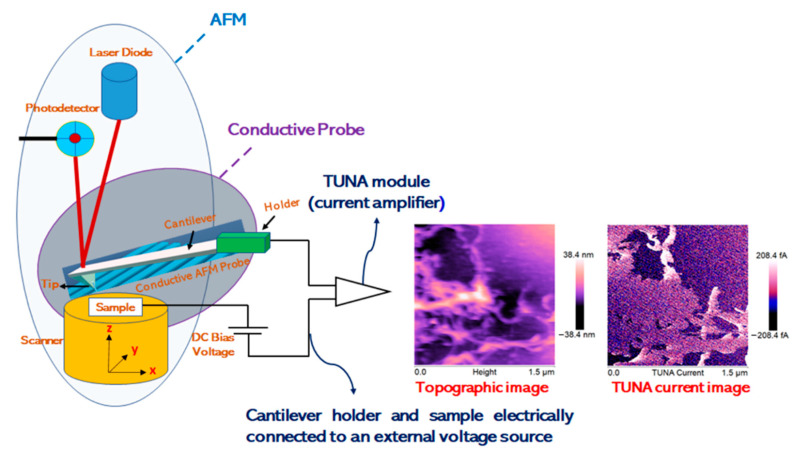
Schematic drawing of TUNA apparatus.

Figure 2 shows the schematic drawing of the Tunneling Atomic Force Microscopy (TUNA). The TUNA mode needs TUNA Module to be able to measure the electric properties of the analyzed samples. TUNA Module can be easily represented as a current amplifier used for amplification of electric charge acquired from the sample by a conductive AFM probe. The TUNA images were analyzed using the Bruker software Nanoscope Analysis 1.80 (Build R1.126200). To highlight the morphological peculiarities of both unfunctionalized and functionalized MWCNT, as well as their distribution within the polymeric matrix and their affinity with the globular rubber domains, the nanocomposites underwent etching treatment [35] before being investigated by TUNA. It is worth noting that thanks to the possibility of detecting currents and, at the same time, providing details on the fine morphological characteristics of the conductive nanocharge inside the host matrix, the TUNA technique gives helpful further information of the nanoscale material properties.

#### 2.2.4. Self-Repairing Efficiency Measurements

Self-repairing efficiency measurements were carried out by determining the values of both critical fracture load *P_c_* of the virgin samples (*P_cVirgin_*) and the healed samples after 24 h (*P_cHealed_*) for the following two nanocomposites: TCTBD+0.5%MWCNT-b and TCTBD+0.5%MWCNT-t. These values have been obtained by the Tapered Double Cantilever Beam (TDCB) fracture tests performed on the developed supramolecular self-responsive nanocomposites, following a procedure reported in our previously published work [35]. This procedure was found to be very effective to assess the self-repairing functionality. It has been demonstrated that the ratio between *P_cHealed_* and *P_cVirgin_* allows the calculation of the healing efficiency of the sample, according to the following equation:

(1) $$\eta =\frac{P_{cHealed}}{P_{cVirgin}}\times 100$$

## 3. Results and Discussions

### 3.1. FTIR Investigation of Modified Carbon Nanotubes

FTIR analysis of modified carbon nanotubes was conducted with the aim of identifying the functional groups bonded to the MWCNT, thus demonstrating the success of the performed covalent modification.

After the functionalization procedure [35], two kinds of ligands, namely barbiturate and thymine-based ligands, indicated with the acronyms *b* and *t*, respectively, have been covalently bonded to MWCNT.

These ligands present different types of functional groups, such as carbonyl (C=O), azido (N≡N), methyl (-CH_3_-), or methylene (-CH_2_-) groups, which have been identified by infrared spectroscopy.

**Figure 3:**
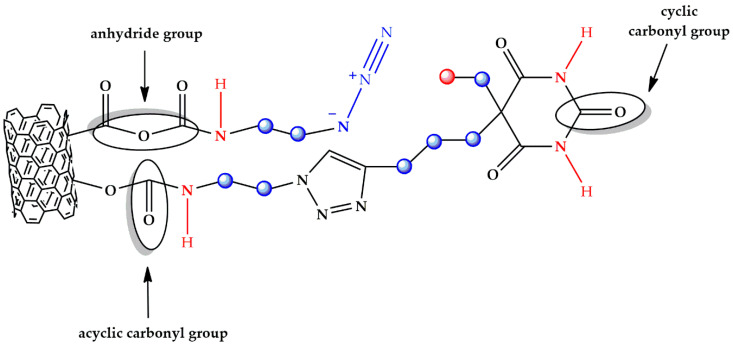
Chemical structure of MWCNT-b with the characteristic functional groups highlighted.

**Figure 4:**
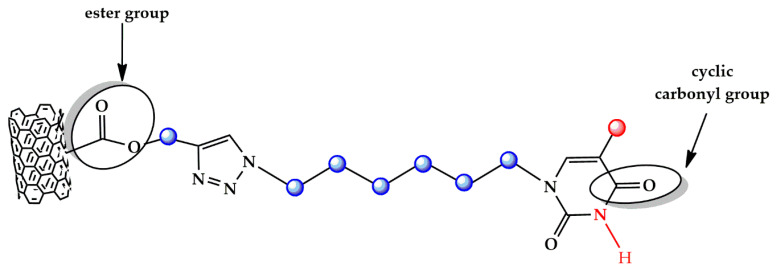
Chemical structure of MWCNT-t with the characteristic functional groups highlighted.

Figure 3 and Figure 4, which represent the chemical structures of MWCNT-b and MWCNT-t, respectively, highlight these characteristic functional groups.

**Figure 5:**
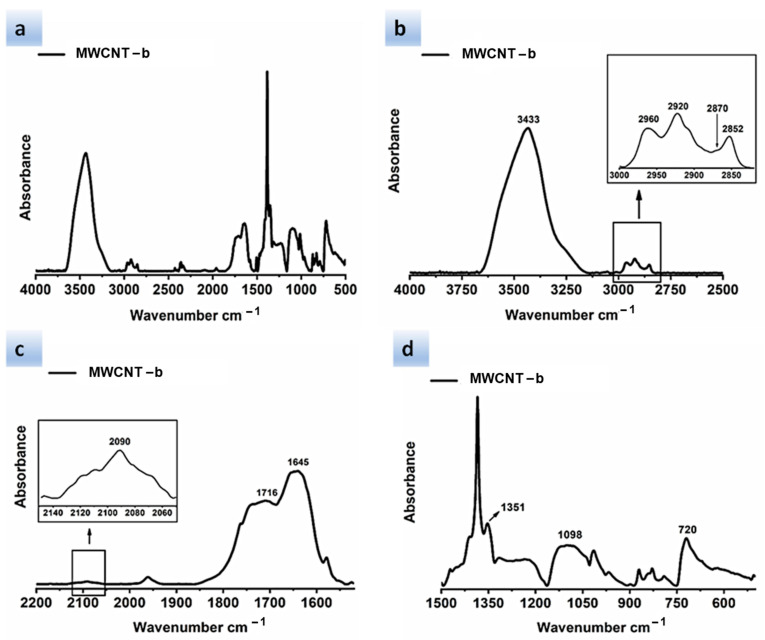
FTIR spectrum of MWCNT–b shown at different spectral ranges of wavelengths: (**a**) spectral range 4000–500 cm^−1^; (**b**) spectral range 4000–2500 cm^−1^ with inset at higher magnification; (**c**) spectral range 2200–1550 cm^−1^ with inset at higher magnification; (**d**) spectral range 1500–525 cm^−1^.

Figure 5 shows different spectral ranges of wavelengths for the FTIR spectrum of the MWCNT-b. In particular, Figure 5a shows the spectral range 4000–500 cm^−1^, Figure 5b shows the spectral range 4000–2500 cm^−1^ with inset at higher magnification, Figure 5c shows the spectral range 2200–1550 cm^−1^ with inset at higher magnification, and Figure 5d shows the spectral range 1500–525 cm^−1^. The band centered at around 3433 cm^−1^ (see spectrum of Figure 5b) can be attributed to the stretching vibration of the secondary amide group N-H [38], highlighted in red in Figure 3, while the absorptions arising in the wavenumber range between 3000 and 2850 cm^−1^ belong to the C-H stretching vibrations. In particular, the band centered at about 2960 cm^−1^ belongs to the C-H asymmetrical (ν_as_) stretching of the methyl group (CH_3_—represented with a red sphere in Figure 3), while the bands occurring at about 2852 and 2920 cm^−1^ result from the symmetrical and asymmetrical stretching vibrations of the methylene group, respectively (CH_2_—represented with a blue sphere in Figure 3). Furthermore, it is visible in the less intense shoulder band at about 2870 cm^−1^, belonging to CH_3_ symmetrical stretching. It is worth noting that asymmetrical and symmetrical vibrations for methylene appear in the spectrum within a range of ±10 cm^−1^. The band detected at about 720 cm^−1^ (Figure 5d) results from methylene bending vibrations and, in particular, it corresponds to the rocking vibration (ρ) of the CH_2_ group [39,40].

In the spectrum of Figure 5c, the band visible at about 2090 cm^−1^ is attributable to the asymmetric stretching vibration of the azido group N≡N, highlighted in blue in Figure 3. The corresponding azido group N≡N symmetric stretching vibration is located at a considerably lower frequency of 1351 cm^−1^ (Figure 5d).

In general, this band is not only much weaker, but also appears to be more variable in position [41]. The broad peak that appears at about 1716 cm^−1^ (Figure 5c) is associated with the acyclic carbonyl group indicated in Figure 3, while the band positioned at about 1645 cm^−1^ can be attributed to the C=O stretching vibration of the cyclic carbonyl group shown in Figure 3, considering that, usually, the carbonyl group of δ-lactams (a cyclic amide with a six-atom ring) occurs in the infrared range around 1660 cm^−1^ [42].

In Figure 5d, the broad band located at about 1098 cm^−1^ is attributed to the stretching vibration of the CO-O-CO anhydride group, visible in Figure 3 [43]. Furthermore, in this region the band due to the C-N stretching vibration of azido derivatives can fall [44].

**Figure 6:**
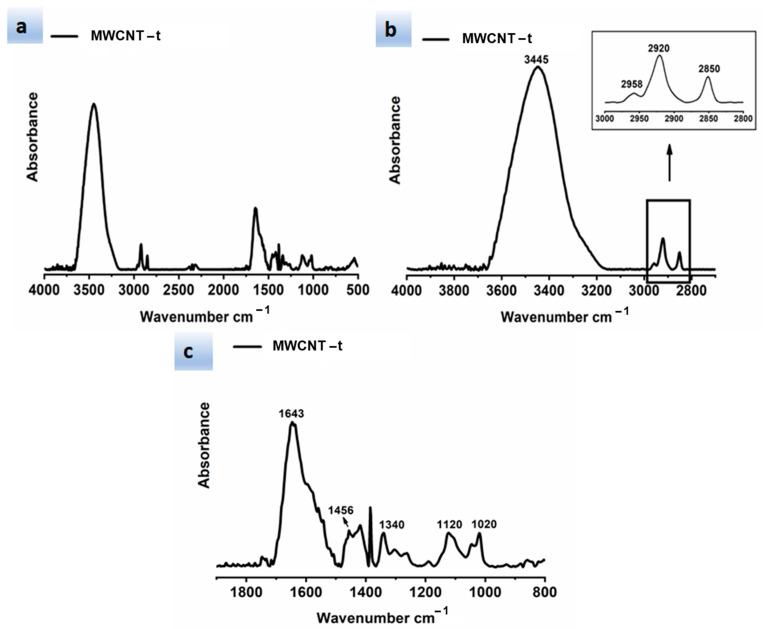
FTIR spectrum of MWCNT–t shown at different spectral ranges of wavelengths: (**a**) spectral range 4000–500 cm^−^^1^; (**b**) spectral range 4000–2700 cm^−1^ with inset at higher magnification; (**c**) spectral range 1900–800 cm^−1.^

Figure 6 shows different spectral ranges of wavelengths for the FTIR spectrum of the MWCNT-t. In particular, Figure 6a–c show the following spectral ranges of 4000–500 cm^−1^, 4000–2700 cm^−1^ with inset at higher magnification, and 1900–800 cm^−1^, respectively.

Figure 6b clearly shows the bands located at 2958, 2920, and 2850 cm^−1^, which are associated with the C-H asymmetrical and symmetrical stretching of methyl and methylene groups (highlighted by red and blue spheres in Figure 4, respectively).

In particular, the bands located at 2920 and 2850 cm^−1^ belong to the -CH_2_ asymmetric and symmetric stretching, respectively, while the asymmetric CH_3_ stretching shows a less intense band at 2958 cm^−1^ [40].

It is worth noting that the higher intensity of the band at about 2920 cm^−1^ is most probably due to the higher number of -CH_2_- groups present in the sample MWCNT-t with respect to the sample MWCNT-b. In the spectra of MWCNT-t, it is also possible to observe the amide stretching vibration of the amide group N-H (highlighted in red in the Figure 4) at 3445 cm^−1^ (Figure 6b) and the C=O stretching vibration of cyclic carbonyl groups at 1643 cm^−1^ (Figure 6c).

The spectrum in Figure 6c also shows the C-H bending vibrations of the methylene group, such as rocking at around 1456 cm^−1^, and twisting and wagging vibrations around 1340 cm^−1^ [39].

Finally, the two bands at about 1120 and 1020 cm^−1^ can be attributed to the C-O stretching vibrations of the ester group (Figure 6c), consisting of two asymmetrical coupled vibrations: C-C(=O)-O and O-C-C [43].

### 3.2. HRTEM Analysis of Carbon Nanofillers

**Figure 7:**
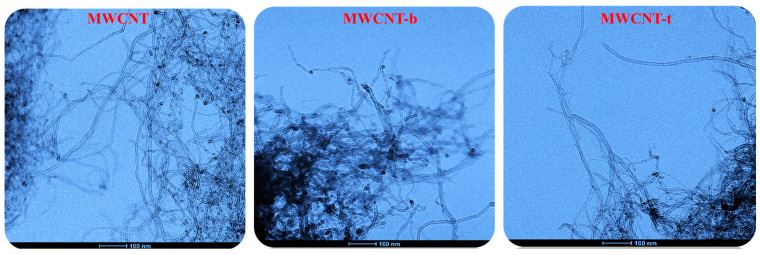
HRTEM pictures of MWCNT, MWCNT-b, and MWCNT-t nanoparticles.

The HRTEM images in Figure 7, captured at the same 100 μm scale bar value, seem to show a reduced length of the functionalized nanotubes compared to that of the unfunctionalized ones.

Furthermore, the presence of functional groups on the MWCNT walls seems to favor the assembly among the MWCNTs.

This peculiarity is a direct consequence of the covalent functionalization carried out on the carbon nanotubes. In this regard, it should be noted that the covalent functionalization acts above all in the areas where the carbon nanofiller has more defects associated with amorphous carbon originated from the MWCNTs synthesis, thus leading to a modification of MWCNT structural quality linked to the aspect ratio, or the length-to-diameter ratio [34,45].

In fact, acid treatments used to carry out covalent functionalization also cause the shortening of MWCNT length [35,46,47], which is unfavorable for applications dependent on higher aspect ratios, for instance the manufacturing of polymeric composites [48,49]. This effect could be responsible for the strong decrease in the electrical conductivity of the self-repairing samples filled with modified carbon nanotubes.

### 3.3. TUNA Analysis of Carbon Nanotube-Based Epoxy Samples

**Figure 8:**
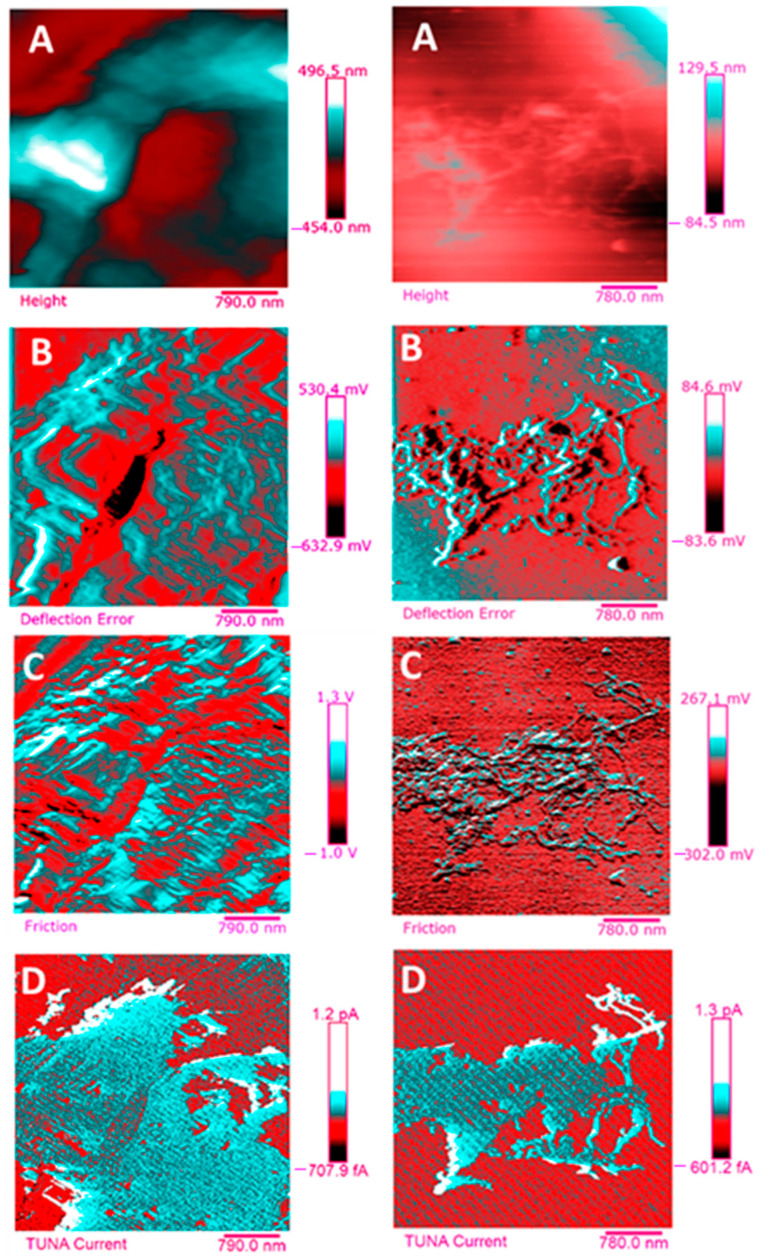
TUNA pictures (2D modality): (**A**) Height; (**B**) Deflection Error; (**C**) Friction; (**D**) TUNA Current of TCTBD+2%MWCNT–b (on the left) and TCTBD+2%MWCNT–t (on the right).

**Figure 9:**
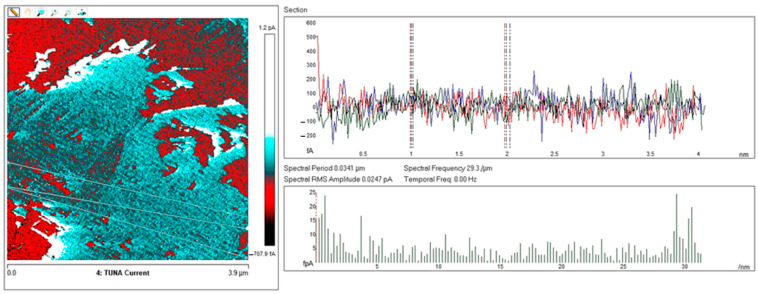
From left to right, TUNA Current picture and current variation profile of the sample TCTBD+2%MWCNT–b.

**Figure 10:**
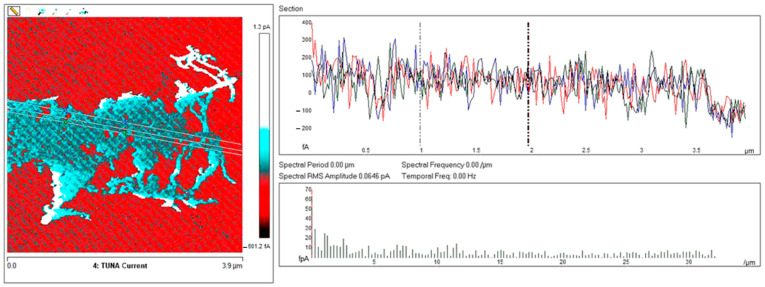
From left to right, TUNA Current picture and current variation profile of the sample TCTBD+2%MWCNT–t.

**Figure 11:**
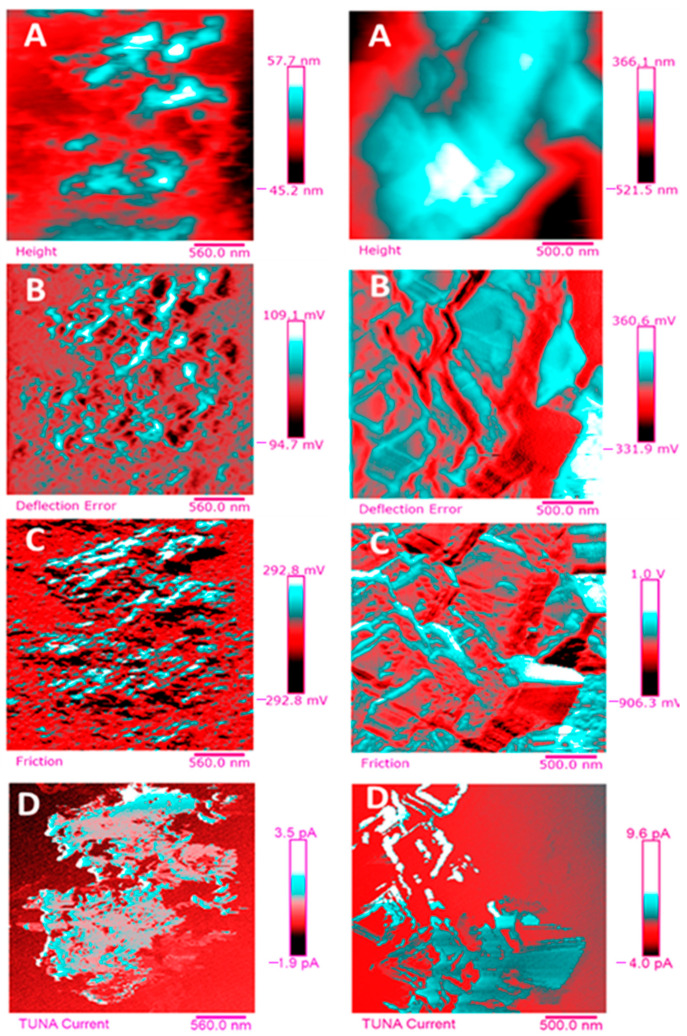
TUNA pictures (2D modality): (**A**) Height; (**B**) Deflection Error; (**C**) Friction; (**D**) TUNA Current of TCTBD+0.5%MWCNT-b (on the left) and TCTBD+0.5%MWCNT-t (on the right).

**Figure 12:**
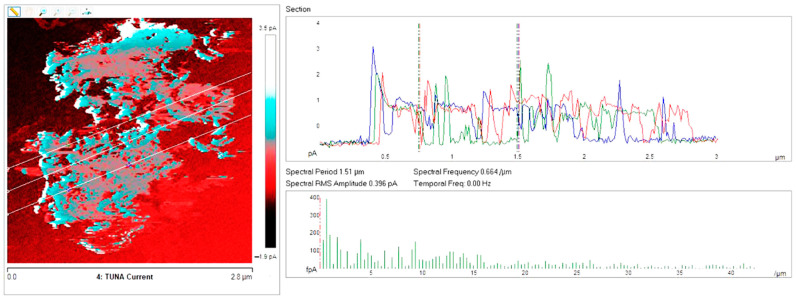
From left to right, TUNA Current picture and current variation profile of the sample TCTBD+0.5%MWCNT-b.

**Figure 13:**
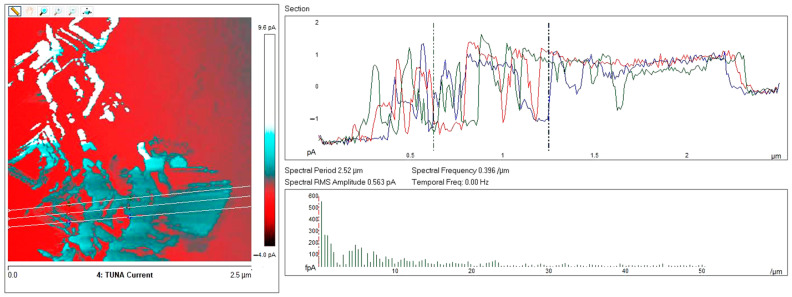
From left to right, TUNA Current picture and current variation profile of the sample TCTBD+0.5%MWCNT-t.

**Figure 14:**
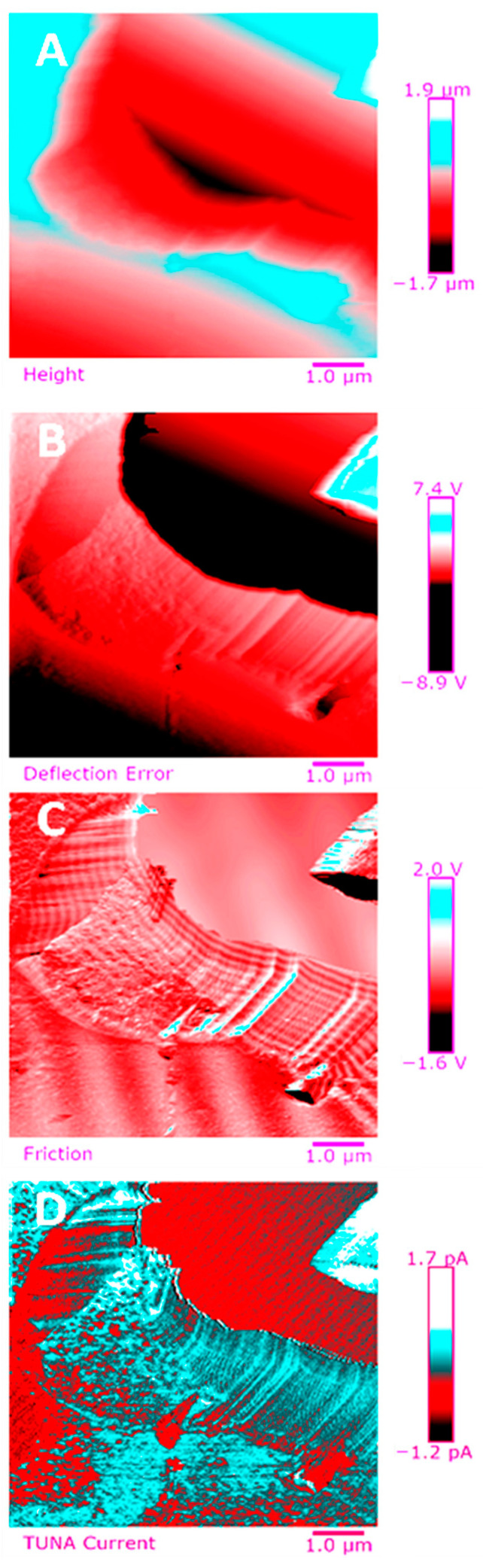
TUNA pictures (2D modality): (**A**) Height; (**B**) Deflection Error; (**C**) Friction; (**D**) TUNA Current of TCTBD+0.5%MWCNT.

**Figure 15:**
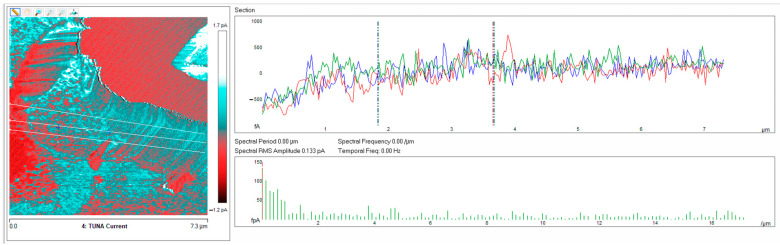
From left to right, TUNA Current picture and current variation profile of the sample TCTBD+0.5%MWCNT.

**Figure 16:**
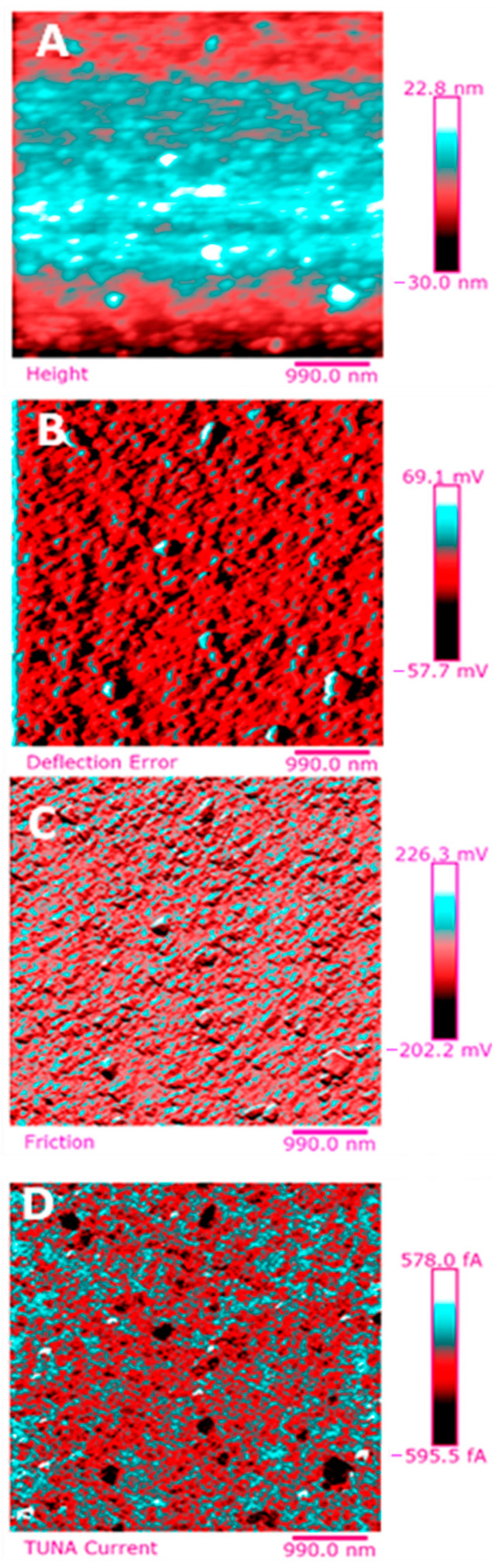
TUNA pictures (2D modality): (**A**) Height; (**B**) Deflection Error; (**C**) Friction; (**D**) TUNA Current of TCTBD+0.5%MWCNT.

**Figure 17:**
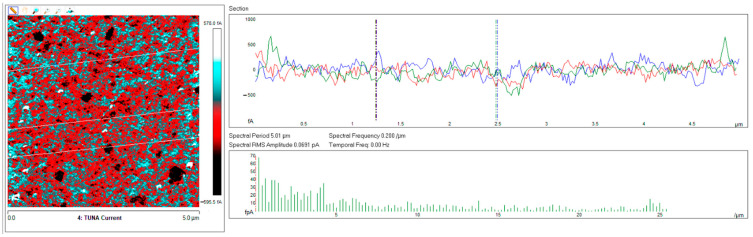
From left to right, TUNA Current picture and current variation profile of the sample TCTBD+0.5%MWCNT.

Below, the results of the morphological characterization through TUNA analysis of the nanocomposites, obtained by homogeneously dispersing the functionalized MWCNT-b and MWCNT-t at two different filler amounts (2 wt% and 0.5 wt%) in the rubber-toughened epoxy formulation, are reported. For comparison, the nanocomposite filled with 0.5 wt% unfunctionalized MWCNT was also analyzed. In this work, the TUNA technique is used to better understand the electrical properties of the conductive nanoparticles embedded in designed advanced materials in light of the DC conductivity results, previously obtained on the same systems [34]. Figure 8 shows eight different TUNA pictures in 2D modality, corresponding to TCTBD+2%MWCNT–b sample (see Heigh (A); Deflection Error (B); Friction (C); and TUNA Current (D) images on the left) and TCTBD+2%MWCNT–t sample (see Heigh (A); Deflection Error (B); Friction (C); and TUNA Current (D) images on the right). It is worth noting that, through the TUNA current pictures, the dispersion state of the carbonaceous nanofillers within the epoxy nanocomposites was evaluated. Figure 9 and Figure 10 show TUNA Current picture and current variation profile of TCTBD+2%MWCNT–b and TCTBD+2%MWCNT–t samples, respectively. Figure 11 shows eight different TUNA pictures in 2D modality, corresponding to TCTBD+0.5%MWCNT–b sample (see Heigh (A); Deflection Error (B); Friction (C); and TUNA Current (D) images on the left) and TCTBD+0.5%MWCNT–t sample (see Heigh (A); Deflection Error (B); Friction (C); and TUNA Current (D) images on the right). Figure 12 and Figure 13 show TUNA Current picture and current variation profile of TCTBD+0.5%MWCNT–b and TCTBD+0.5%MWCNT–t samples, respectively. Figure 14 shows four different TUNA pictures in 2D modality, corresponding to TCTBD+ 0.5%MWCNT sample. Figure 15 shows TUNA Current picture and current variation profile of TCTBD+ 0.5%MWCNT sample. The four different TUNA pictures in 2D modality of TCTBD+0.5%MWCNT are shown in Figure 16. TUNA Current picture and current variation profile of TCTBD+ 0.5%MWCNT sample are shown in Figure 17. (For Figure 8, Figure 11, Figure 14 and Figure 16, the corresponding 3D modality TUNA pictures are shown in Figures S2–S5 inserted in the supplementary electronic materials.) The morphological characteristics of the fracture surface of the nanocomposites are easily observable from the four types of TUNA images, each of which contributes together with the others to outline clear and distinctive information of the sample examined. From the accentuated color contrast that evolves from the darkest color to the brighter color, as it can be easily seen from the scale bar shown for each of the four types of TUNA images, we can obtain significant information on the topographic and electrical properties and on the correlation between them. In this regard, in all the TUNA Current pictures, we can observe that the most sparkling colors identify more conductive regions due to the presence of carbon nanotubes, both unfunctionalized ones and functionalized ones, which are distributed to form a continuous conductive network. The important aspect in the TUNA characterization consists in detecting that, in the TUNA Current images, the most conductive areas of the sample characterized by the brightest colors which, on the scale bar, indicate higher values of current, coincide with the topographic peculiarities (found in the other TUNA images) relating to the conductive nanofiller and the intimate interfacial interaction of it with the host polymeric matrix.

The similarity of the four different TUNA drawings indisputably indicates that the TUNA analysis was carried out successfully, thus allowing the achievement of a concomitant tracing of both the topographic and electrical characteristics of the formulated multifunctional samples.

This was accomplished by employing regulated, reduced forces on the tip during the image acquisition phase.

Functionalized carbon nanotubes are less conductive than unfunctionalized ones so that, although the conductive network is formed in the resin, the electrical conductivity values of nanocomposites containing MWCNT-b and MWCNT-t at 0.5 wt % are lower than those containing pristine MWCNT at the same load percentage, while, on the other hand, by increasing the percentage of load up to 2 wt %, the electrical conductivity of the nanocomposites reaches values similar to those obtained with 0.5 wt % of pristine MWCNT [37]. In particular, for the samples loaded at 2 wt % of MWCNT-b and MWCNT-t, DC electrical conductivity values equal to 6.76 × 10^−3^ and 3.70 × 10^−2^ S/m [34,37], respectively, were recorded. These values are comparable with that of 2.56 × 10^−2^ S/m, obtained by loading the TCTBD resin with 0.5 wt % of pristine MWCNT [34,37].

At the same 0.5 wt %, the nanocomposites based on functionalized MWCNT-b and MWCNT-t, instead, showed very low DC electrical conductivity values equal to 6.28 × 10^−12^ and 6.47 × 10^−12^ S/m, respectively [34,37].

The chosen filler load percentage of 0.5 wt % was found to be above the Electrical Percolation Threshold (EPT) for unfunctionalized MWCNT-based epoxy samples [50]. The insulating behavior shown instead by the nanocomposites filled with the same amount (0.5 wt %) of modified carbon nanotubes is explainable as it has been shown that the functionalization procedure involves the reduction of the electrical conductivity of the nanofiller due to its changed aspect ratio, as evident in the HRTEM pictures of MWCNT-b, and MWCNT-t nanoparticles of Figure 7.

An increase in the weight percentage of nanofillers up to 2 wt % (which in practice match to a load of 1.5 wt % of functionalized MWCNT, estimated by removing the weight of the functional groups) [34] was decisive in determining a substantial increase in the electrical conductivity of the nanocomposites loaded with MWCNT-b and MWCNT-t.

Downstream of the results obtained on the good electrical and self-healing performance of the modified carbon nanotube-based epoxy samples, we can certainly assert that functionalization played a key role in obtaining a self-responsive material.

In fact, it represented a fair compromise in order to realize multifunctional nanocomposites characterized by good thermal, electrical, mechanical, and self-healing properties through an appropriate modulation of the modified nanocharge amount in the range between 0.5 wt % and 2 wt %.

**Figure 18:**
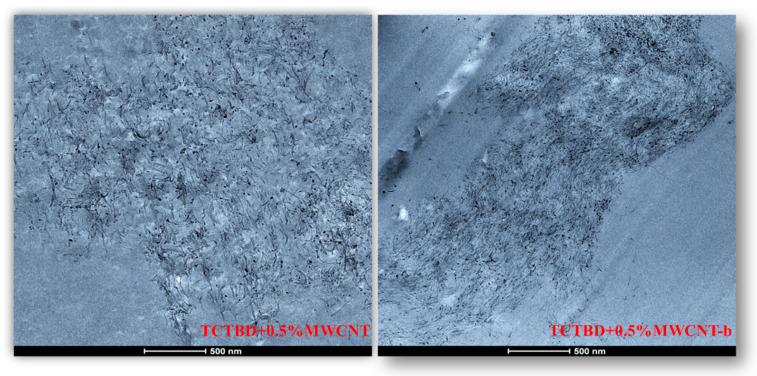
HRTEM pictures of the samples filled with 0.5wt % of MWCNT and MWCNT-b.

In this work, through the TUNA analysis, we have shown that, even in nanocomposites containing 0.5 wt % of modified carbon nanotubes who exhibited isolating behavior, the reduced electrical conductivity of functionalized carbon nanotubes does not prevent the formation of conductive networks (located in different areas of the sample fracture surface), highlighted by the light blue color in TUNA pictures (Figure 11 and Figure S3), where the nanofillers are strongly interconnected with each other and also with the host matrix. In fact, the presence of conductive interconnections, clearly visible mostly in TUNA Current images (Figure 11D and Figure S3D), allows the detection of high current signals at a nanoscale level in the range between 1.9 and 3.5 pA for the sample TCTBD+0.5%MWCNT-b (Figure 11D and Figure S3D on the left) and between 4.0 and 9.6 pA for the sample TCTBD+0.5%MWCNT-t (Figure 11D and Figure S3D on the right). These current values are high even at 0.5 wt %, which is the load percentage at which the functionalized carbon nanotube-based samples investigated in this work exhibit insulating behavior. For the highest load percentage (2 wt %) of functionalized nanofillers, the 2D and 3D TUNA Current images of TCTBD+2%MWCNT-b (Figure 8D and Figure S2D on the left) and TCTBD + 2%MWCNT-t (Figure 8D and Figure S2D on the right) allow the detection of current values between 707.9 fA to 1.2 pA, and between 601.2 fA to 1.3 pA, respectively. From all TUNA images (Figure 8 and Figure S2), it is possible to discriminate through the strong contrast of colors detectable on the sidebar an awesome distribution of the functionalized nanofillers that appear to be intimately interconnected and, by virtue of their strong affinity with the rubber phase uniformly dispersed in the polymeric matrix, bring together the rubber and the matrix. A denser and more extensive conductive network in nanocomposites containing the highest percentage of 2 wt % of MWCNT-b and MWCNT-t, appropriately chosen with the purpose of increasing the electrical performance of self-repairing nanocomposites loaded with 0.5 wt % of the same nanofillers, is clearly distinguishable in the bright light blue color. Obviously, it is important to underline that the strong oxidizing etching solution which is highly corrosive towards the resin has allowed the nanofillers to emerge on the sample surface, thus effectively highlighting the presence of the nanofillers and conductive nanodomains. In all TUNA pictures of the epoxy samples loaded with 0.5 wt % of MWCNT-b (Figure 11 and Figure S3 on the left) and MWCNT-t (Figure 11 and Figure S3 on the right), even if, especially in the TUNA Current images (Figure 11D and Figure S3D), the local electrical nanodomains are characterized by carbon nanotubes that interpenetrate each other and organize themselves to form conductive networks visible on the analyzed surface. In some points of the surface, a lower number of conductive nanoparticles is observed, which can be explained with the lower load percentage of 0.5 wt %. From the comparison with the fracture surface of the sample TCTBD+0.5%MWCNT (Figure 16 and Figure S5), we can see how, at the same percentage of 0.5 wt % of unfunctionalized MWCNT (endowed with a higher intrinsic electrical conductivity than functionalized MWCNT-b and MWCNT-t, since the covalent functionalization somewhat decreases the π-delocalization of the sp^2^ electrons of the carbon nanotubes), the conductive interweaving of carbon nanotubes firmly bound to the host polymeric matrix appears much more compact and continuous, crossing the entire investigated area of the sample compared to the samples filled at the same amount of 0.5 wt % of modified carbon nanotubes. The results obtained by mapping the electrical nanodomains of both unfunctionalized and functionalized carbon nanotube-based samples are in perfect agreement with their characteristic electrical behavior. It is very important to underline how the advanced TUNA technique, precisely by virtue of the fact that it acts at the nanoscale level, has proved extremely impactful in this work, and also in detecting the effect that functionalization has had on the globular domains of rubber. The functionalized carbon nanotubes can cause the rupture of the globular domains of the rubber. The latter, in fact, appear less evident by observing the TUNA images relating to the samples based on MWCNT-b and MWCNT-t nanofillers (Figure 11 and Figure S3). The spherical domains of the rubber phase are instead more evident in the sample loaded with unfunctionalized MWCNT, as it can easily be seen in the TUNA images shown in Figure 16 and Figure S5. In particular, the TUNA Current image (Figure 16D and Figure S5D), whose current values recorded are between 595.5 and 578 fA, detects the presence of the rubber domains that appear rounded and clearly distinguishable within the conductive network formed by the carbon nanotubes bound together and with the polymer matrix. The rubber spherical regions, in some points of the investigated area, appear separated by a continuous net of a brighter conductive phase. In fact, the detection of some dark brown rubber domains that seem to be isolated on the sample surface is most likely due to the oxidizing action of the etching procedure which breaks the bonds between the rubber circles that emerge more on the surface and the epoxy matrix, thus locally determining a decrease in the values of the electric current, which turns out to be of the order of femtoampere (fA). Through the mapping of the conductive nanodomains of the investigated nanocomposites offered by the TUNA Current images, as previously mentioned, we can evaluate the dispersion level of the conductive nanoparticles within the polymeric matrix. In this regard, from left to right, the TUNA Current picture and current variation profile of the samples TCTBD+2%MWCNT-b (Figure 9), TCTBD+2%MWCNT-t (Figure 10), TCTBD+0.5%MWCNT-b (Figure 12), TCTBD+0.5%MWCNT-t (Figure 13), and TCTBD+0.5%MWCNT (Figure 15 and Figure 17) are shown. The current variations of the nanocomposites identified by the colors green, red, and blue correspond to the three white lines that have been drawn on each TUNA Current image and which indicate the position where the current variation profiles were taken. The somewhat regular frequency of the fluctuations attributable to nanofiller/matrix interchanges along the three lines proves the good distribution reached for the samples TCTBD+2%MWCNT-b (Figure 9) and TCTBD+2%MWCNT-t (Figure 10), where evident current variations, such as to justify the high electrical conductivity for these samples loaded with 2 wt % of functionalized carbon nanotubes, can be found. In the case of the samples TCTBD+0.5%MWCNT-b (Figure 12) and TCTBD+0.5%MWCNT-t (Figure 13), a greater number of relative irregularities compared to the samples filled with 2 wt % of functionalized nanofillers is observable. These irregularities are very presumably attributable to the fact that, although functionalized carbon nanotubes are arranged to form a more extensive and continuous network, as shown in HRTEM pictures of the sample TCTBD+0.5%MWCNT and TCTBD+0.5%MWCNT-b (Figure 18), by virtue of the hydrogen bonding moieties on MWCNT walls, however, compared to what happens in the presence of unfunctionalized MWCNT (Figure 18), in some points, the conductive nanoparticles MWCNT-b and MWCNT-t are present in smaller quantities due to the low weight percentage (0.5 wt %) of nanofiller inside the epoxy matrix. In fact, as already said before, these samples were found to be below the Electrical Percolation Threshold (EPT) at the 0.5 wt % of MWCNT-b and MWCNT-t [37]. Figure 15 and Figure 17 show the TUNA Current picture (on the left) and current variation profile (on the right) of the sample TCTBD+0.5%MWCNT. We can observe that, for this sample, the conductive nanoparticles are well distributed in the polymeric matrix. This can be deduced from the regular frequency of the current variations (along the three white lines) due to the nanofiller/matrix sequence (Figure 15 and Figure 17).

### 3.4. Assessment of Self-Repairing Functionality

**Figure 19:**
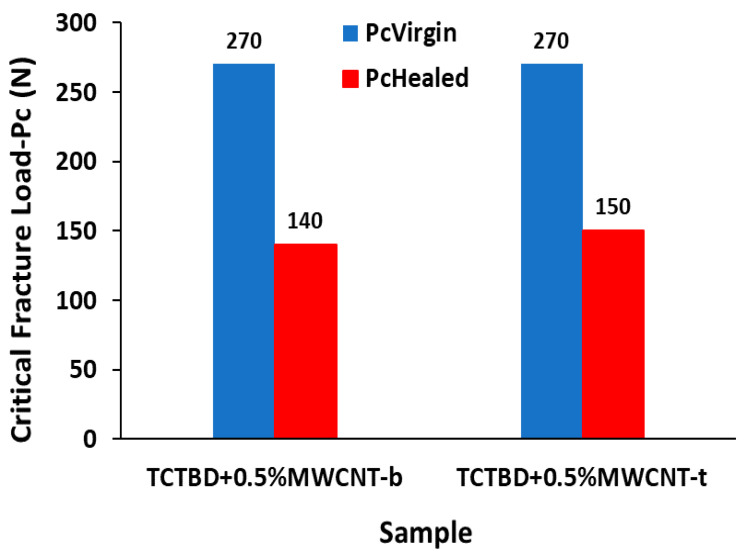
Histogram showing the values of both the critical fracture load Pc of the virgin samples (PcVirgin) and the healed samples (PcHealed) for the two nanocomposites: TCTBD+0.5%MWCNT-b and TCTBD+0.5%MWCNT-t subjected to the TDCB fracture test.

In order to demonstrate the self-repairing functionality of the investigated supramolecular self-responsive nanocomposites, data before and after damage were here provided. In particular, a histogram that reports the values of both critical fracture load Pc of the virgin samples (PcVirgin) and the healed samples (PcHealed) for the two nanocomposites TCTBD+0.5%MWCNT-b and TCTBD+0.5%MWCNT-t is shown in Figure 19. Self-repairing efficiencies (η) have been calculated using Equation (1), as described in Section 2.2.4. From the PcVirgin and PcHealed values reported in the histogram for the samples TCTBD+0.5%MWCNT-b and TCTBD+0.5%MWCNT-t, self-repairing efficiency values of 52% and 56% have been found, respectively [35].

## 4. Conclusions

In this work, the novel TUNA technique has proven to be a powerful tool in revealing the morphology on the nanoscale of both unfunctionalized and functionalized carbon nanotube-based conductive networks and detecting the electrical current values on the same location for direct correlation. Furthermore, TUNA Current images, through the analysis of the frequency of the current changes due to nanofiller/matrix alternations, also allow the studying of the state of the dispersion of the functionalized and unfunctionalized nanofiller. TUNA pictures highlight that the presence of functional groups on the carbon nanotube walls seems to favor the assembly among MWCNTs, as it can be also deduced by HRTEM images (and, even more, from the TUNA pictures). This result is not surprising because reversible hydrogen bridging bonds are established not only between functional groups on MWCNTs and functional groups of the polymeric matrix, but also between MWCNTs and MWCNTs through the presence of donor and acceptor groups on the wall of carbon nanotubes. When we compare unfunctionalized and functionalized carbon nanotubes, the nature of the functional groups plays a determining role on the dispersion state. The specific nature of the functionalization here performed determines a greater difficulty in the dispersion of the carbon nanotubes. In fact, although the functional groups tend to weaken the Van der Waals interactions due to steric factors related to the spacing of the MWCNTs, in our case, other different attractive interactions between the carbon nanotubes come into play.

It is worth noting that this study has been performed using the same times and the same methodology for the carbon nanotube dispersion (unfunctionalized and functionalized) to better analyze the effect of the performed functionalization. For the future, such an investigation could help factorize the contributions to healing efficiency due to the reversible attractive interactions between carbon nanotubes (MWCNTs—MWCNTs) and carbon nanotubes and matrix (MWCNTs –EPOXY).

This manuscript, for the first time, through TUNA analysis, provides clear evidence of such a problem. The above-mentioned aspects cannot be clearly investigated through traditional macroscopic electrical measurements.

## Data Availability

Not applicable.

## Supplementary Materials

The following are available online at https://www.mdpi.com/article/10.3390/polym13091401/s1. Figure S1. Drawing of the supramolecular network in the sample loaded with barbiturate functionalized MWCNT-b; Figure S2. TUNA pictures (3D modality): (A) Heigh; (B) Deflection Error; (C) Friction; (D) TUNA Current of TCTBD+2%MWCNT-b (on the left) and TCTBD+2%MWCNT-t (on the right); Figure S3. TUNA pictures (3D modality): (A) Heigh; (B) Deflection Error; (C) Friction; (D) TUNA Current of TCTBD+0.5%MWCNT-b (on the left) and TCTBD+0.5%MWCNT-t (on the right); Figure S4. TUNA pictures (3D modality): (A) Heigh; (B) Deflection Error; (C) Friction; (D) TUNA Current of TCTBD+0.5%MWCNT; Figure S5. TUNA pictures (3D modality): (A) Heigh; (B) Deflection Error; (C) Friction; (D) TUNA Current of TCTBD+0.5%MWCNT.
