# Supplementary material for: Tunneling Atomic Force Microscopy Analysis of Supramolecular Self-Responsive Nanocomposites
Source: Polymers (Basel). 2021 Apr 26;13(9):1401. doi: 10.3390/polym13091401 (PMC8123594; doi:10.3390/polym13091401)
Supplement: Supplementary file 1 [file polymers-13-01401-s001.zip › polymers-1163614-supplementary.pdf]

Supplementary electronic materials

Tunneling Atomic Force Microscopy analysis of supramolecular self-responsive nanocomposites

Marialuigia Raimondo [1,*], Elisa Calabrese [1], Wolfgang H. Binder [2], Philipp Michael [2], Sravendra Rana [3] and Liberata Guadagno [1]

1 Department of Industrial Engineering, University of Salerno, Via Giovanni Paolo II, 132, 84084, Fisciano (SA), Italy; mraimondo@unisa.it (M.R.); elicalabrese@unisa.it (E.C.); lguadagno@unisa.it (L.G.)

2 Macromolecular Chemistry, Institute of Chemistry, Faculty of Natural Science II, Martin Luther University Halle-Wittenberg, Von-Danckelmann-Platz 4, 06120 Halle, Germany; wolfgang.binder@chemie.uni-halle.de (W.H.B.); philipp.michael@chemie.uni-halle.de (P.M.)

3 Department of Chemistry, University of Petroleum and Energy Studies (UPES), Bidholi Dehradun 248007, India; srana@ddn.upes.ac.in (S.R.)

* Correspondence: mraimondo@unisa.it; Phone: +39 089 964019 (M.R.)

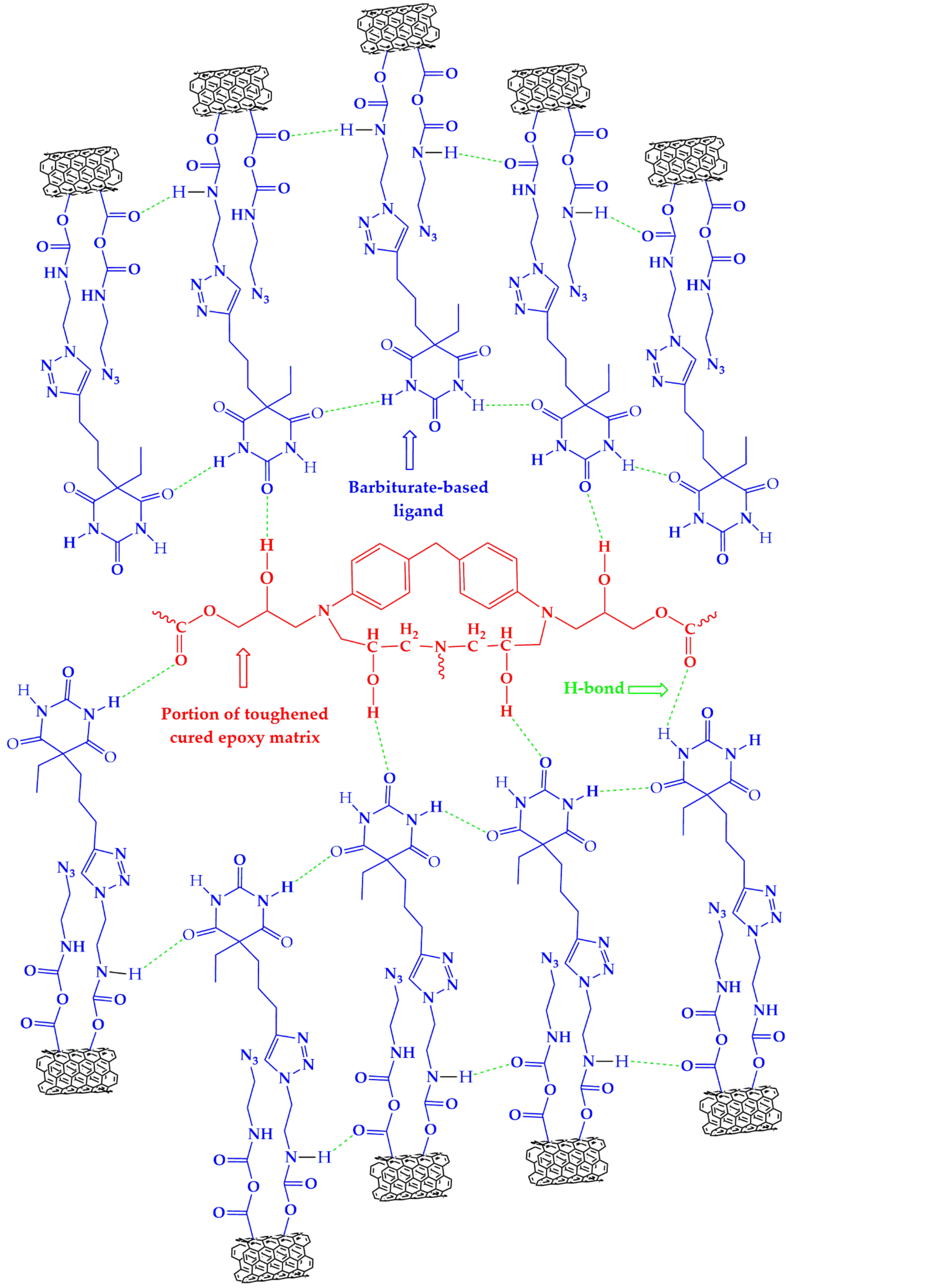

Figure S1. Drawing of the supramolecular network in the sample loaded with barbiturate functionalized MWCNT-b.

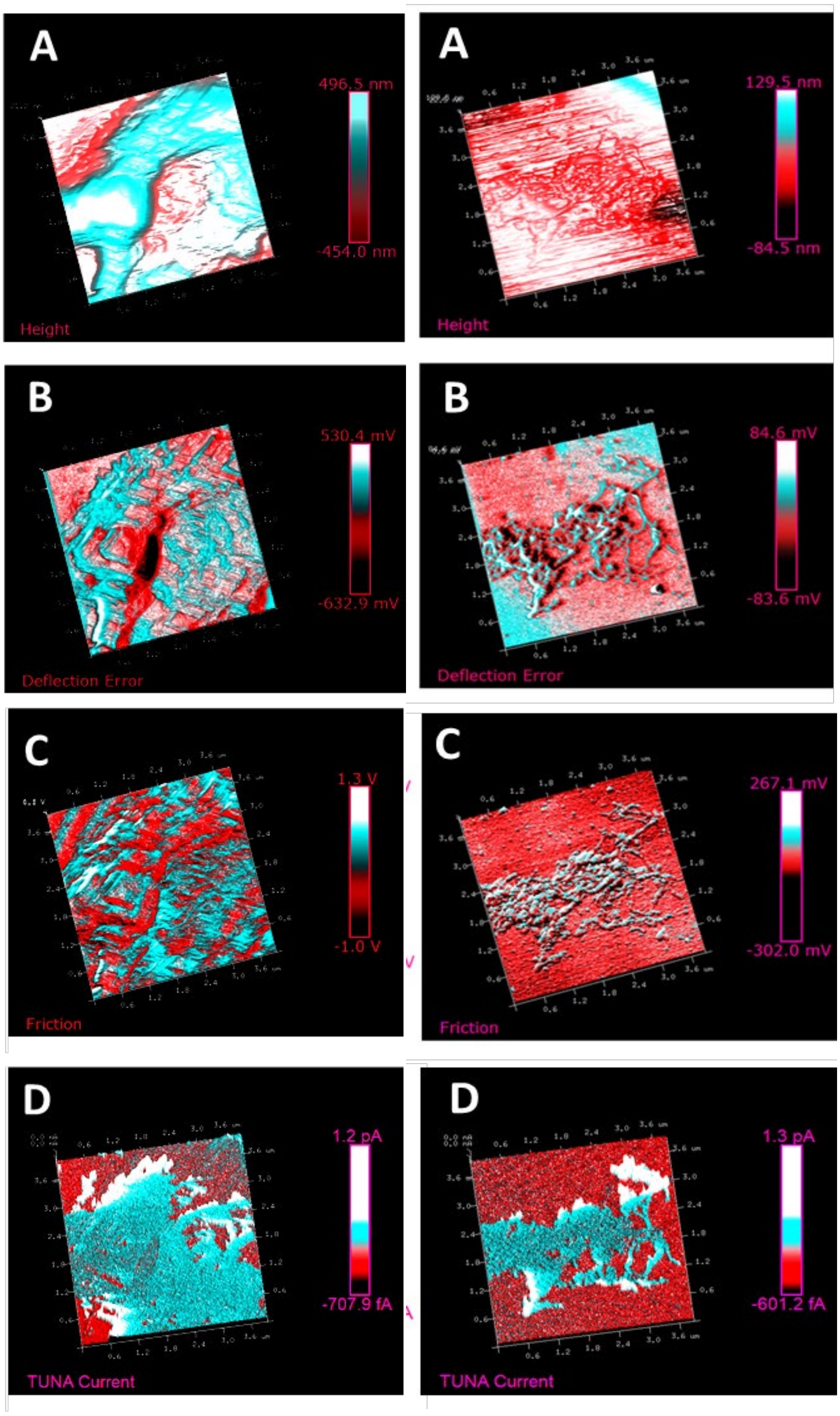

Figure S2. TUNA pictures (3D modality): A) Heigh; B) Deflection Error; C) Friction; D) TUNA Current of TCTBD+2%MWCNT-b (on the left) and TCTBD+2%MWCNT-t (on the right).

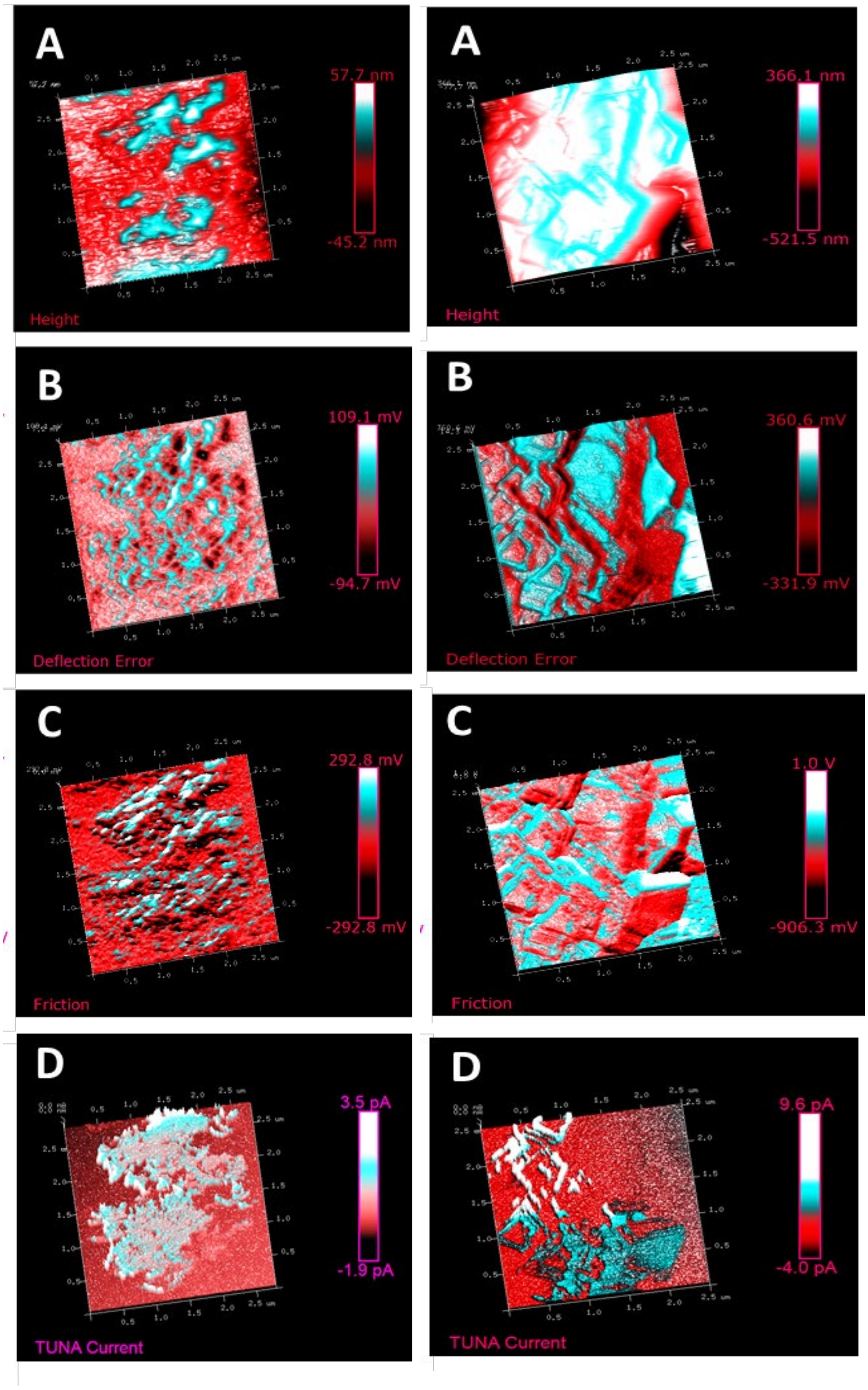

Figure S3. TUNA pictures (3D modality): A) Heigh; B) Deflection Error; C) Friction; D) TUNA Current of TCTBD+0.5%MWCNT-b (on the left) and TCTBD+0.5%MWCNT-t (on the right).

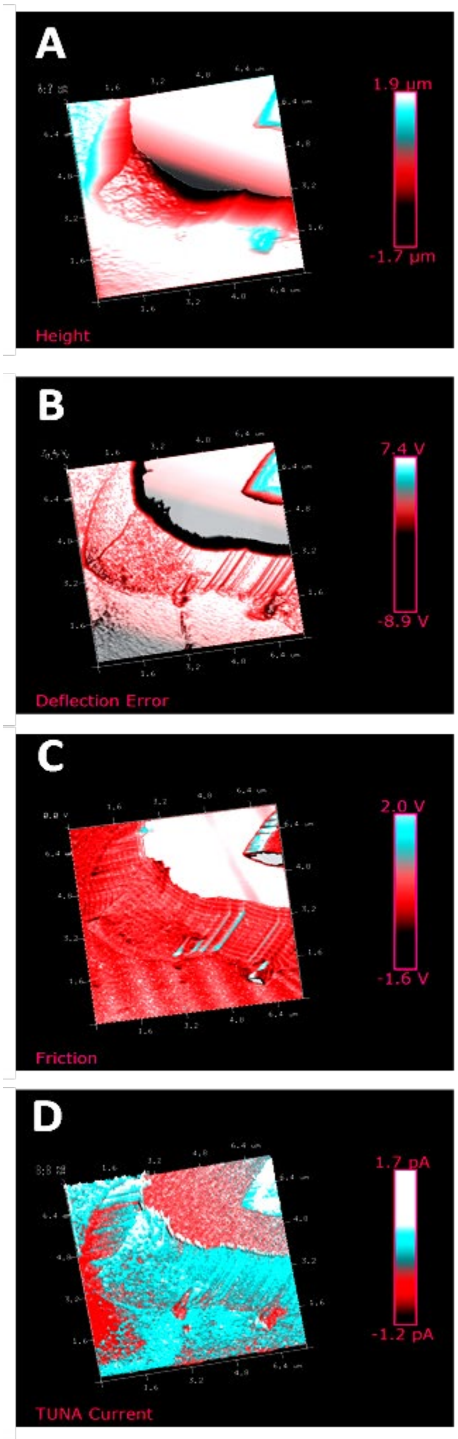

Figure S4. TUNA pictures (3D modality): A) Heigh; B) Deflection Error; C) Friction; D) TUNA Current of TCTBD+0.5%MWCNT.

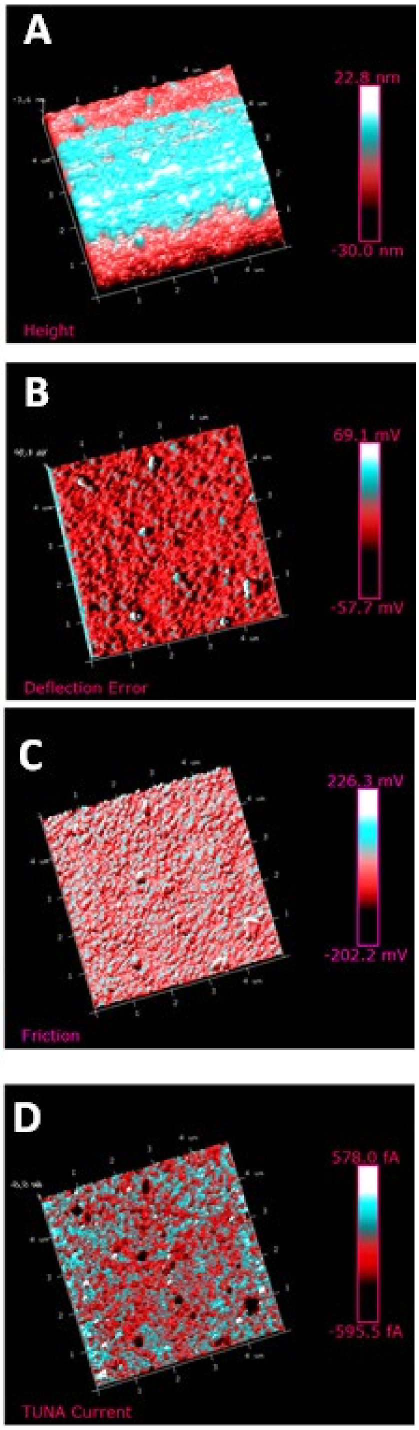

Figure S5. TUNA pictures (3D modality): A) Heigh; B) Deflection Error; C) Friction; D) TUNA Current of TCTBD+0.5%MWCNT.
